# Independent movement of the voltage sensors in K_V_2.1/K_V_6.4 heterotetramers

**DOI:** 10.1038/srep41646

**Published:** 2017-01-31

**Authors:** Elke Bocksteins, Dirk J. Snyders, Miguel Holmgren

**Affiliations:** 1 Laboratory for Molecular Biophysics, Physiology and Pharmacology, Department for Biomedical Sciences, University of Antwerp, Antwerp, Belgium; 2 Molecular Neurophysiology Section, Porter Neuroscience Research Center, National Institute of Neurological Disorders and Stroke, National Institutes of Health, Bethesda, Maryland, USA

## Abstract

Heterotetramer voltage-gated K^+^ (K_V_) channels K_V_2.1/K_V_6.4 display a gating charge-voltage (Q_V_) distribution composed by two separate components. We use state dependent chemical accessibility to cysteines substituted in either K_V_2.1 or K_V_6.4 to assess the voltage sensor movements of each subunit. By comparing the voltage dependences of chemical modification and gating charge displacement, here we show that each gating charge component corresponds to a specific subunit forming the heterotetramer. The voltage sensors from K_V_6.4 subunits move at more negative potentials than the voltage sensors belonging to K_V_2.1 subunits. These results indicate that the voltage sensors from the tetrameric channels move independently. In addition, our data shows that 75% of the total charge is attributed to K_V_2.1, while 25% to K_V_6.4. Thus, the most parsimonious model for K_V_2.1/K_V_6.4 channels’ stoichiometry is 3:1.

Voltage-gated K^+^ (K_V_) channels contribute significantly to the excitability of several cell types, including neurons and cardiac myocytes. They regulate the resting membrane potential, the membrane repolarization and the action potential shape and firing frequency^1^. K_V_ channels perform these roles by opening, closing and inactivating upon changes in membrane potential. They function as tetramers of α-subunits. Each subunit contains six transmembrane segments. The first four (S1–S4) form a structural domain called the voltage sensing domains (VSDs), which as the name implies, is responsible for sensing transmembrane voltage^2^. Charged residues in the S4 transmembrane segment form the main voltage sensing components^3^,^4^,^5^,^6^. The last two transmembrane segments (S5–S6) of each α-subunit arrange to form a central ion conducting pore^4^. Upon membrane depolarization, the S4 segments move upwards via a combined rotating, tilting and vertical displacement which can be recorded as gating currents (I_Q_)^2^. These conformational changes are transmitted via an electromechanical coupling to an intracellular channel gate allowing channels to open^7^,^8^,^9^,^10^,^11^. This intracellular gate is formed by the C-terminal ends of the four S6 transmembrane segments which obstruct the central ion conducting pore via a bundle crossing formation when channels are closed^12^,^13^,^14^. In many K_V_ channels, sustained depolarizations induce a slow inactivation that involves changes within the selectivity filter resulting in a non-conductive state^15^,^16^,^17^. In some cases, slow inactivation can develop even before opening of the intracellular channel gate, a process known as closed-state inactivation^18^.

Based on sequence homology, the Shaker-related K_V_ channel subunits are divided into eight subfamilies: K_V_1-K_V_6 and K_V_8-K_V_9^19^. Members of the K_V_5, K_V_6, K_V_8 and K_V_9 subfamilies are collectively called “silent” subunits because they do not form functional homotetramer channels at the plasma membrane, but they assemble with K_V_2 subunits to form functional heterotetramers^20^. Fluorescence Resonance Energy Transfer (FRET) experiments suggest that, in case of K_V_2.1/K_V_9.3, heterotetramerization occurs with a 3:1 (K_V_2.1:K_V_9.3) stoichiometry^21^. Heterotetramers, like K_V_2.1/K_V_6.4 channels, display distinct functional properties when compared to K_V_2.1 homotetramers. They have a ~40 mV shifted voltage dependence of inactivation to more negative potentials, a ~5–10 fold reduced current density, a ~2 fold shallower voltage dependence of activation and a more complex activation time course^22^. Interestingly, the gating charge-voltage distribution (Q_V_) of K_V_2.1/K_V_6.4 channels contains two components, whereas the Q_V_ distribution of K_V_2.1 homotetramers displayed only one component^23^. Here, we set to determine the origins of these components in K_V_2.1/K_V_6.4 heterotetramers. We determined the voltage dependence of the rates of chemical modification of cysteines within the S4 transmembrane segments of K_V_6.4 and K_V_2.1 and compared them with the gating charge distribution. Our results show that the more negative component of the Q_V_ distribution, which carries ~25% of the total charge, originates from the movement of the voltage sensors of K_V_6.4 subunits, while the remaining ~75% of the charge corresponds to the movement of the VSDs of the K_V_2.1 subunits. Therefore, the VSDs of subunits K_V_2.1 and K_V_6.4 within a heterotetramer channel move independently and they likely assemble with a stoichiometry of 3:1 (K_V_2.1: K_V_6.4).

## Results

### MTSET modification and charge displacements of K_V_2.1(V296C) homotetramers and K_V_2.1(V296C)/K_V_6.4 heterotetramers

**Figure 1:**
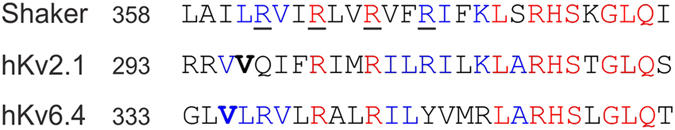
Sequence alignment of the Shaker, K_V_2.1 and K_V_6.4 S4 region. The underlined arginine residues in Shaker represent those that contribute to the gating charge. The bold valine residues were substituted for cysteines in K_V_2.1 (K_V_2.1(V296C)) and K_V_6.4 (K_V_6.4(V335C)). In red are shown residues that are conserved among the three channel sequences, while blue residues represent those conserved in only two sequences. The remaining ones are shown in black.

**Figure 2:**
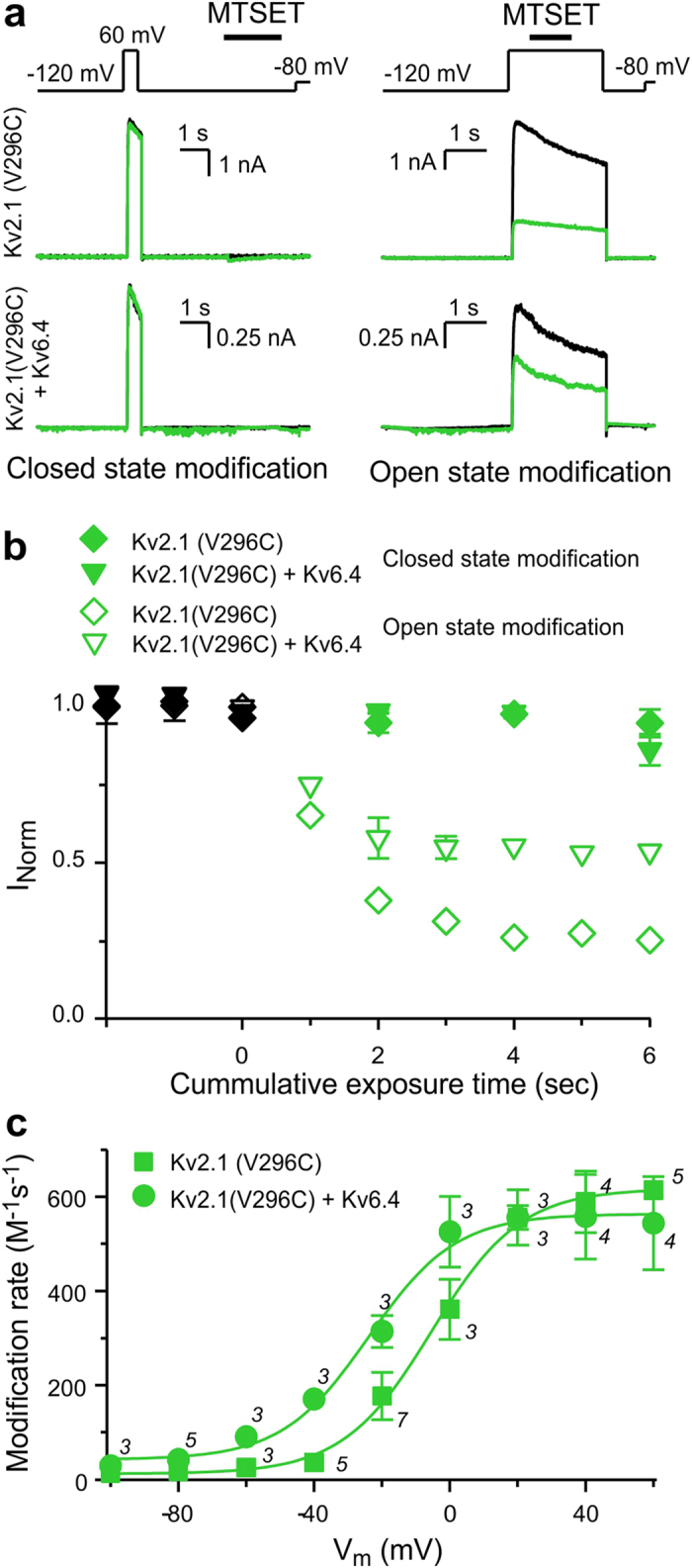
MTSET modification of K_V_2.1 subunits. (**a**) Representative current recordings to determine whether K_V_2.1(V296C) homotetramers and K_V_2.1(V296C)/K_V_6.4 heterotetramers are modified by MTSET in the closed state (left) or open state (right). The applied pulse protocols and modification are given on top. (**b**) Time course of modification. Symbols represent normalized current reductions by MTSET modifications of K_V_2.1(V296C) (diamond) and K_V_2.1(V296C)/K_V_6.4 (triangle down) channels at +60 mV (open symbols) and −120 mV (closed symbols). Black symbols denote normalized values before modification (**c**) Voltage dependence of the modification rate of K_V_2.1(V296C) (square) and K_V_2.1(V296C)/K_V_6.4 (circle). The solid lines represent sigmoidal fits. The best-fit parameter values for V_1/2_ for the K_V_2.1(V296C) and K_V_2.1(V296C)/K_V_6.4 data were −5.4 mV and −23.9 mV, respectively. Numbers above symbols represent the number of cells analyzed at each voltage. Data are represented as the mean ± SEM (shown when it is larger than the size of the symbol).

To assess the origin of the gating charge components of the K_V_2.1/K_V_6.4 heterotetramers’ Q_V_ distribution, we first substituted V296 of K_V_2.1, located at the external end of the S4 transmembrane segment, by a cysteine (Fig. 1). This cysteine was used as target for state dependent chemical modification using the membrane-impermeant thiol reagent MTSET^24^, in both homotetramers and as heterotetramers with WT K_V_6.4 (Fig. 2). Applications of 1 mM MTSET during depolarizing pulses to 60 mV (open state) reduced the K_V_2.1(V296C) and K_V_2.1(V296C)/K_V_6.4 current amplitudes to approximately 25% and 50% of their initial value, respectively (Fig. 2a,b). In contrast, similar MTSET exposures during hyperpolarizing pulses to −120 mV (closed state) reduced the current amplitudes of K_V_2.1(V296C) and K_V_2.1(V296C)/K_V_6.4 channels by only 5% (Fig. 2a,b). These current reductions were similar to the one observed after similar MTSET applications on open and closed WT K_V_2.1 homotetramers and K_V_2.1/K_V_6.4 heterotetramers (Supplementary Fig. 1).

Application of MTSET in the open state did not only affect the current density of K_V_2.1(V296C) and K_V_2.1(V296C)/K_V_6.4 channels but also their biophysical properties (Supplementary Fig. 2). To assess the voltage dependence of modification, we determined MTSET modification rates between −100 mV and 60 mV (with 20-mV increments) (Fig. 2c). The modification rates for both K_V_2.1(V296C) homotetramers and K_V_2.1(V296C)/K_V_6.4 heterotetramers followed sigmoidal distributions with voltage (Fig. 2c).

**Figure 3:**
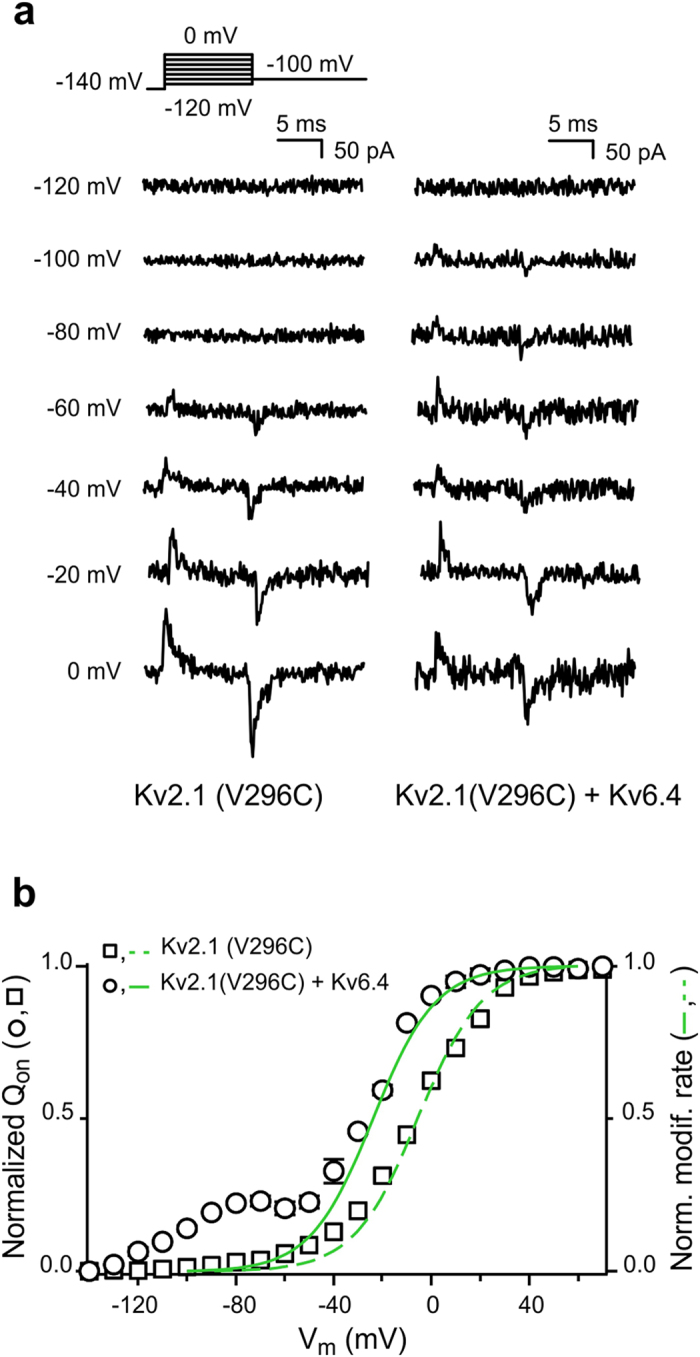
Gating charge displacement in K_V_2.1(V296C) homotetramers and K_V_2.1(V296C)/K_V_6.4 heterotetramers. (**a**) Representative gating current recordings of K_V_2.1(V296C) homotetramers (left) and K_V_2.1(V296C)/K_V_6.4 heterotetramers (right) at −120 mV, −100 mV, −80 mV, −60 mV, −40 mV, −20 mV and 0 mV. The applied pulse protocol is given on top. (**b**) Q_V_ distribution of K_V_2.1(V296C) (square) and K_V_2.1(V296C)/K_V_6.4 (circle) channels. The Q_V_ distributions were obtained by plotting the area under the recorded ON-gating currents as a function of voltage. For comparison, the normalized MTSET modification curves of K_V_2.1(V296C) and K_V_2.1(V296C)/K_V_6.4 (dashed and solid green line, respectively) are shown. Data are represented as the mean ± SEM (shown when it is larger than the size of the symbol).

If the voltage dependence of modification represents changes in accessibility originated by the voltage sensor movements, they should parallel the voltage dependence of the gating charge displacement. Figure 3a shows representative gating current recordings of homotetramers K_V_2.1(V296C) and heterotetramers K_V_2.1(V296C)/K_V_6.4. The Q_V_ distribution of K_V_2.1(V296C) homotetramers could be fitted by a single Boltzmann function with Q_1/2_ = −6.5 ± 0.9 mV (Fig. 3b, squares). Similar to the Q_V_ distribution of WT K_V_2.1/K_V_6.4^23^, K_V_2.1(V296C)/K_V_6.4 heterotetramers displayed a charge distribution with two gating charge components (Q_1/2_ = −109.8 ± 1.8 mV and Q_1/2_ = −21.1 ± 0.9 mV, Fig. 3b, circles). The normalized best-fit of the MTSET modification for K_V_2.1(V296C) channels superimposes their corresponding Q_V_ distribution (Fig. 3b; squares, dashed line), as expected by the voltage dependence of the modification rates originating from changes in accessibility due to movements of the voltage sensor. In the case of K_V_2.1(V296C)/K_V_6.4 heterotetramers, the modification rates align to the larger and more positive gating charge component (Fig. 3b, solid line). These results indicate that the more positive component of the K_V_2.1(V296C)/K_V_6.4 Q_V_ distribution is associated with the movements of the VSD of K_V_2.1(V296C) subunits.

### MTSET modification and charge displacements of K_V_2.1/K_V_6.4(V335C) heterotetramers

**Figure 4:**
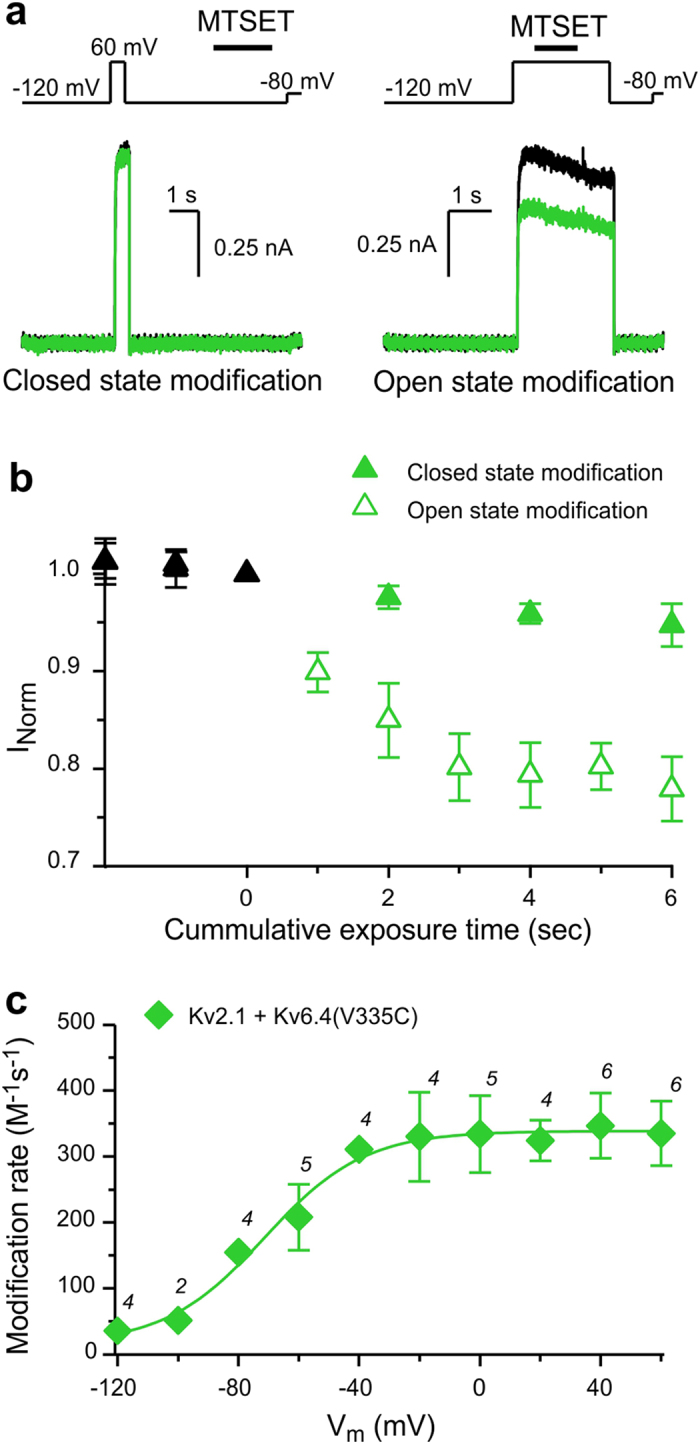
MTSET modification of K_V_6.4 subunits. (**a**) Representative current recordings to determine whether K_V_2.1/K_V_6.4(V335C) channels are modified by MTSET in the closed state (left) or open state (right). The applied pulse and modification protocols are given on top. (**b**) Time course of modification. Symbols represent current normalized to the value at time 0. Open symbols depict MTSET modifications at + 60 mV while closed symbols represent modification at −120 mV. Black symbols symbolize normalized values before modification. (**c**) Voltage dependence of the modification rate of K_V_2.1/K_V_6.4(V335C). Solid line represents a sigmoidal fit. The best-fit parameter values for V_1/2_ and k were −71.3 mV and 16.5, respectively. Numbers above symbols represent the number of cells analyzed at each voltage. Data are represented as the mean ± SEM (shown when it is larger than the size of the symbol).

To assess the voltage dependence of K_V_6.4 subunits we substituted V335 of K_V_6.4, located at the external end of the S4 transmembrane segment, by a cysteine (Fig. 1). Application of 1 mM MTSET on closed K_V_2.1/K_V_6.4(V335C) channels did not significantly affect the current density of these channel constructs (~5%, Fig. 4a,b). In contrast, ionic currents were reduced by ~20% when MTSET was applied during channel opening.

Application of MTSET in the open state also affected the biophysical properties of K_V_2.1/K_V_6.4(V335C) channels (Supplementary Fig. 3), which provides us confidence that the small changes in current density are indeed linked to modification of K_V_6.4(V335C) subunits.

Figure 4c shows the voltage dependence of MTSET modification for K_V_2.1/K_V_6.4(V335C) heterotetramer channels. Notably, at −80 mV in which K_V_2.1/K_V_6.4(V335C) channels are not open yet (Supplementary Fig. 2a), significant chemical modification could be detected with rates of 154.9 ± 16.4 M^−1^s^−1^ (Fig. 4c). This observation suggests that the voltage sensors of K_V_6.4(V335C) are moving before channel opening.

**Figure 5:**
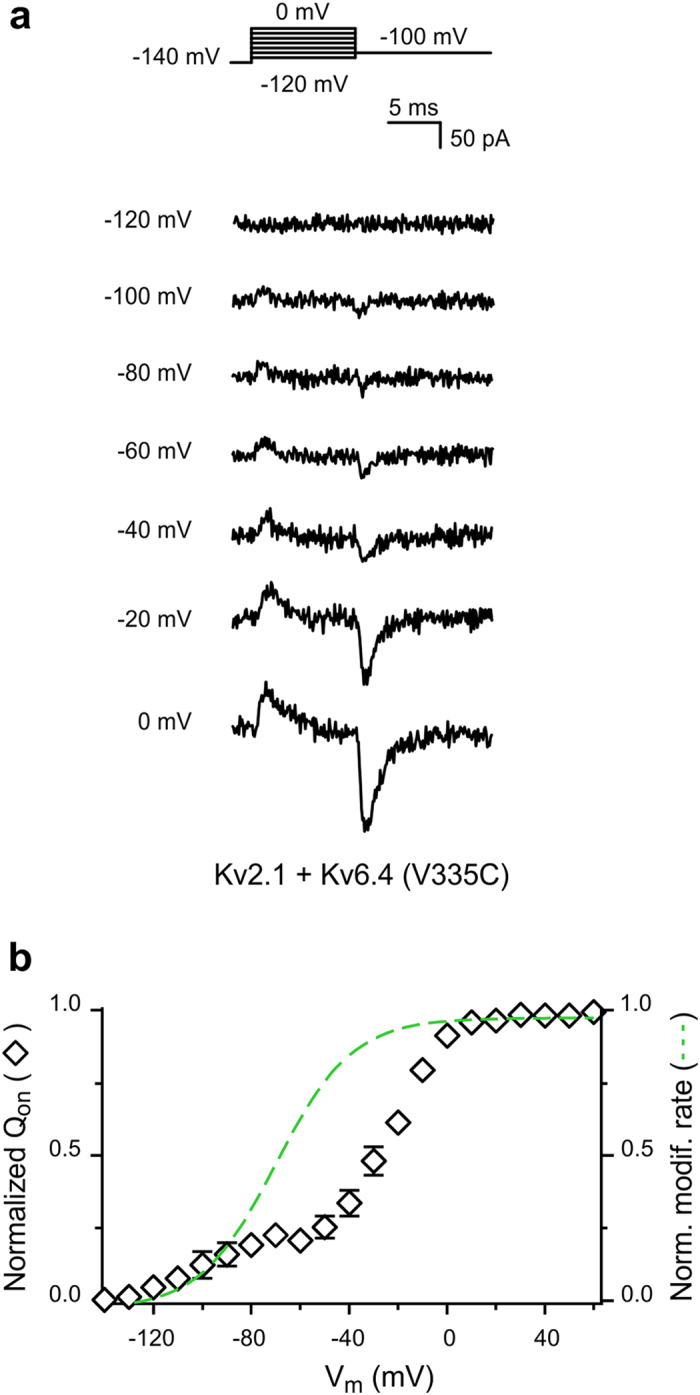
Gating charge displacement in K_V_2.1/K_V_6.4(V335C) channels. (**a**) Representative gating current recordings of K_V_2.1/K_V_6.4(V335C) channels at −120 mV, −100 mV, −80 mV, −60 mV, −40 mV, −20 mV and 0 mV. The applied pulse protocol is given on top. (**b**) Q_V_ distribution of K_V_2.1/K_V_6.4(V335C) channels. The Q_V_ distribution was determined as described in Fig. 3b. For comparison, the normalized MTSET modification curve (dashed green line) is shown. Data are represented as the mean ± SEM (shown when it is larger than the size of the symbol).

Next, we asked how the voltage dependence of modification of K_V_6.4 subunits relates to the gating charge movements of K_V_2.1/K_V_6.4(V335C) heterotetramers. Figure 5a shows typical gating current recordings of K_V_2.1/K_V_6.4(V335C) channels at different potentials. These heterotetramer channels also displayed a charge distribution with two components (Fig. 5b; open diamonds). The more negative and smaller component is characterized by Q_1/2_ = −103.8 ± 7.7 mV whereas the more positive and larger component displayed a Q_1/2_ = −21.6 ± 2.0 mV (n = 4). Unequivocally, the voltage dependence of the modification rates (Fig. 5b, dash line) does not parallel the larger and more positive component of the Q_V_ distribution. Nonetheless, these modification rates do not overlap with the voltage dependence of the smaller component of the gating charge distribution. A plausible explanation might be that V335C becomes increasingly accessible at the latest transitions of the K_V_6.4 voltage sensor movements. These results suggest that the more negative component of the K_V_2.1/K_V_6.4(V335C) Q_V_ distribution represent the movement of the VSD of K_V_6.4(V335C) subunits.

## Discussion

By using a variety of approaches, it has been well established that the voltage sensors from voltage-activated channels undergo conformational changes in response to changes in voltage^25^,^26^,^27^,^28^,^29^,^30^,^31^,^32^,^33^,^34^,^35^,^36^,^37^. Here we used state dependent chemical accessibility of cysteines substituted at the external end of the S4 transmembrane segments of K_V_2.1 and K_V_6.4 subunits to understand their contributions in sensing voltage when they form K_V_2.1/K_V_6.4 heterotetramers.

K_V_2.1/K_V_6.4, K_V_2.1(V296C)/K_V_6.4 and K_V_2.1/K_V_6.4(V335C) heterotetramers have Q_V_ distributions that are composed by two well-defined gating charge components. The voltage dependence of chemical modification of K_V_2.1(V296C)/K_V_6.4 heterotetramers superimposes the larger and more positive component of its corresponding Q_V_ distribution. On the contrary, the voltage dependence of modification of K_V_2.1/K_V_6.4(V335C) heterotetramers is left-shifted by more than 40 mV. In fact, the modification rates are about 80% of their maximal values at voltages where the more positive component of the Q_V_ distribution begins to develop. These results combined strongly suggest that the two components of the Q_V_ distributions in heterotetramers arise from the independent movements of the VSDs of the different subunits forming the channel. Interestingly, two well separate components in Q_V_ distributions from homotetramer K_V_ channels have been reported for many mutations within the VSD of Shaker channels^38^,^39^,^40^,^41^. In these cases, however, the most likely explanations for the appearance of a second component in the Q_V_ distributions originate from the kinetic separation of distinct charge movements steps along a sequential kinetic scheme. Even though the voltage sensors from subunits K_V_2.1 and K_V_6.4 appears to move independently, the presence of K_V_6.4 in heterotetramers does have an influence on the gating charge distribution of K_V_2.1 subunit (see Fig. 3b). Consequently, K_V_6.4 incorporation into channel complexes carries new kinetic properties for activation and inactivation providing a mechanism to fine tune cell excitability^42^. Further, K_V_2.1 is abundantly expressed in most human tissues while expression of silent subunits like K_V_6.4 is more restricted^42^; for example, K_V_6.4 can only be detected in motor neurons whereas K_V_2.1 is expressed in both sensory and motor neurons. Therefore, it is conceivable that K_V_2.1/K_V_6.4 channels tune cellular excitability in a tissue-specific manner^42^. Indeed, K_V_6.4 malfunction decreased neuromuscular output generated by fast motor neurons^43^,^44^ and disturbed spermiogenesis^45^, whereas K_V_6.4 gene variations has been linked to migraine^46^ and changes in the formation of the brain ventricular system^47^.

Heterotetramer channels composed of two different subunits can assemble either as a dimer of dimers leading to a 2:2 stoichiometry^48^ or a 3:1 stoichiometry^21^. Our data shows that 75% of the total charge is attributed to K_V_2.1, while 25% to K_V_6.4. If we assume that subunits K_V_2.1 and K_V_6.4 contribute with the similar amount of gating charge per subunit, our results indicate that the most parsimonious model for K_V_2.1/K_V_6.4 channels’ stoichiometry will be 3:1.

## Methods

### Channel constructs and mutagenesis

Human K_V_2.1 in the peGFP-n1 vector (Clontech), human K_V_6.4 in the peGFP-n1 vector (Clontech) and human K_V_6.4 in the RBG4 vector were constructed as previously described^23^,^49^. Human K_V_2.1 was inserted in the mammalian expression vector RBG4 (kindly provided by J.S. Trimmer, UC Davis, CA, USA). To this end, a second PstI RE-site was introduced in the hK_V_2.1 in peGFP-n1 clone and hK_V_2.1 was subcloned in the RBG4 vector via a PstI (New England Biolabs) RE-digest. To obtain higher expression, the UTR of the rK_V_2.1 in RBG4 clone was introduced before the hK_V_2.1 coding sequence in the RBG4 vector using reverse PCR technique. Cysteine substitutions in K_V_2.1 and K_V_6.4 were performed using standard PCR techniques and suitable mutant primers. The presence of the desired modifications and the absence of unwanted mutations were confirmed by DNA sequencing.

### DNA expression and cell culture

All channel construct’s DNA were expressed in HEK293 cells using the Lipofectamine3000 reagents (Invitrogen^®^, ThermoFisher Scientific) following the manufacturer’s recommended protocol. HEK293 cells were cultivated in DMEM/F12 (1:1) with L-Glutamine and 2.438 g/l sodium bicarbonate medium supplemented with 10% fetal bovine serum, US origin and 0.1% (10 mg/ml) Gentamicin reagent solution (all purchased from Gibco ^®^, ThermoFisher Scientific) at 37 °C under a humidified, 5% CO_2_ enriched atmosphere. For ionic current recordings of WT and mutant K_V_2.1 homotetramers, 50 ng of the WT or mutant hK_V_2.1 in peGFP-n1 DNA was transfected whereas ionic current recordings of WT and mutant K_V_2.1/K_V_6.4 heterotetramers were obtained by co-transfecting 0.5 μg of the WT or mutant hK_V_2.1 in peGFP-n1 construct with 5 μg of the WT or mutant hK_V_6.4 in peGFP-n1 clone. Gating current recordings of K_V_2.1 and K_V_2.1/K_V_6.4 channels were obtained by transfecting 1 μg of the WT or mutant hK_V_2.1 in RBG4 clone and by co-transfecting 1 μg of the WT or mutant hK_V_2.1 in RBG4 clone with 2 μg of the WT or mutant hK_V_6.4 in RBG4 clone, respectively. With each transfection, 0.5 μg GFP was co-transfected as a transfection marker. Cells were used for electrophysiological analysis after 1 day (for ionic current recordings) or 2 days (for gating current recordings) of transfection.

### Experimental solutions

The intracellular solution used for ionic current recordings was composed of (in mM): 140 KCl, 3 MgATP, 5 EGTA and 10 HEPES (pH = 7.35 with NaOH), whereas that used for gating current recordings contained (in mM): 140 N-Methyl-D-Glucamine Chloride, 3 MgATP, 5 EGTA and 10 HEPES (pH = 7.35 with HCl). The extracellular solution used to record ionic currents contained (in mM): 145 NaCl, 4 KCl, 1.8 CaCl_2_, 1 MgCl_2_, 10 HEPES and 10 glucose (pH = 7.2 with NaOH), whereas that for gating currents was comprised of (in mM): 140 Tetraethyl Ammonium Chloride, 1 KCl, 1.8 CaCl_2_, 1 MgCl_2_, 10 HEPES and 10 glucose (pH = 7.2 with N-Methyl-D-Glucamine Chloride). All chemical were purchased from Sigma-Aldrich. To perform the MTSET experiments, a fresh stock solution of 100 mM MTSET was daily made by dissolving [2-(Trimethylammonium)ethyl]methanotiosulfonate Bromide (Toronto Research Chemicals) in nuclease-free water and stored on ice during use. A 1 mM work solution was made just before MTSET application by diluting the MTSET stock solution in the extracellular recording solution.

### Electrophysiological recordings

Both ionic and gating current recordings were obtained at room temperature (20–22 °C) from whole cells, using an Axopatch-200B amplifier (Axon Instruments) connected to a Digidata 1440 data acquisition system (Axon Instruments). Command voltages were controlled using the pClamp10 software (Axon Instruments) and recordings were sampled at 10 kHz and low-pass filtered at 1 kHz. Patch pipettes were pulled with a P-97 micropipette puller (Sutter Instrument Company) from 1.5 mm borosilicate glass (Harvard apparatus) and heat polished. Patch pipettes were filled with an intracellular solution and cells were continuously superfused with an extracellular solution (see “Experimental Solutions”). MTSET was applied by using a computer-controlled solenoid-based perfusion system. Cells used to obtain the MTSET data were positioned in front of the perfusion system in such manner that they were only exposed to either the control extracellular solution or the MTSET-containing extracellular solution. Correct pipette positioning for MTSET application and the rate of perfusion change was verified after each experiment by a computer-controlled switch between the control extracellular solution and a 2-fold diluted extracellular solution. Cells were excluded from analysis if voltage errors exceeded 5 mV after series resistance compensation.

## Additional Information

**How to cite this article**: Bocksteins, E. *et al*. Independent movement of the voltage sensors in K_V_2.1/K_V_6.4 heterotetramers. *Sci. Rep.* **7**, 41646; doi: 10.1038/srep41646 (2017).

**Publisher's note:** Springer Nature remains neutral with regard to jurisdictional claims in published maps and institutional affiliations.

## Supplementary Material

Supplementary Figures
