# Supplementary material for: Independent movement of the voltage sensors in KV2.1/KV6.4 heterotetramers
Source: Sci Rep. 2017 Jan 31;7:41646. doi: 10.1038/srep41646 (PMC5282584; doi:10.1038/srep41646)
Supplement: Supplementary Figures [file srep41646-s1.pdf]

Independent movement of the voltage sensors in KV2.1/KV6.4 heterotetramers.

Authors: Elke Bocksteins, Dirk J. Snyders, Miguel Holmgren

SUPPLEMENTARY MATERIAL

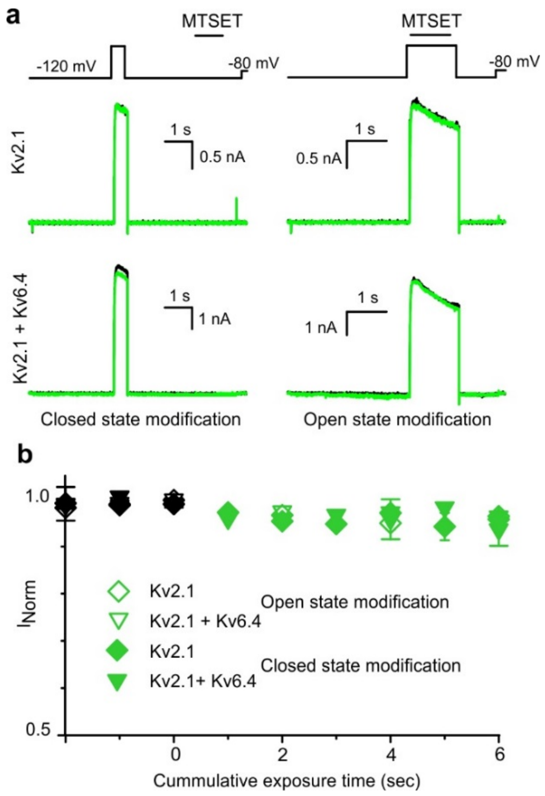

Supplementary Figure 1. MTSET modification of WT Kv2.1 homotetramers and WT Kv2.1/Kv6.4 heterotetramers. (**a**) Representative current recordings to determine whether KV2.1 and Kv2.1/Kv6.4 channels are modified by MTSET in the closed state (left) or open state (right). The applied pulse and modification protocols are given on top. (**b**) Time course of modification. Symbols represent current normalized to the value at time 0. Open symbols depict MTSET modifications at +60 mV while closed symbols represent modification at -120 mV. Black symbols symbolize normalized values before modification. Data are represented as the mean ± SEM (shown when it is larger than the size of the symbol).

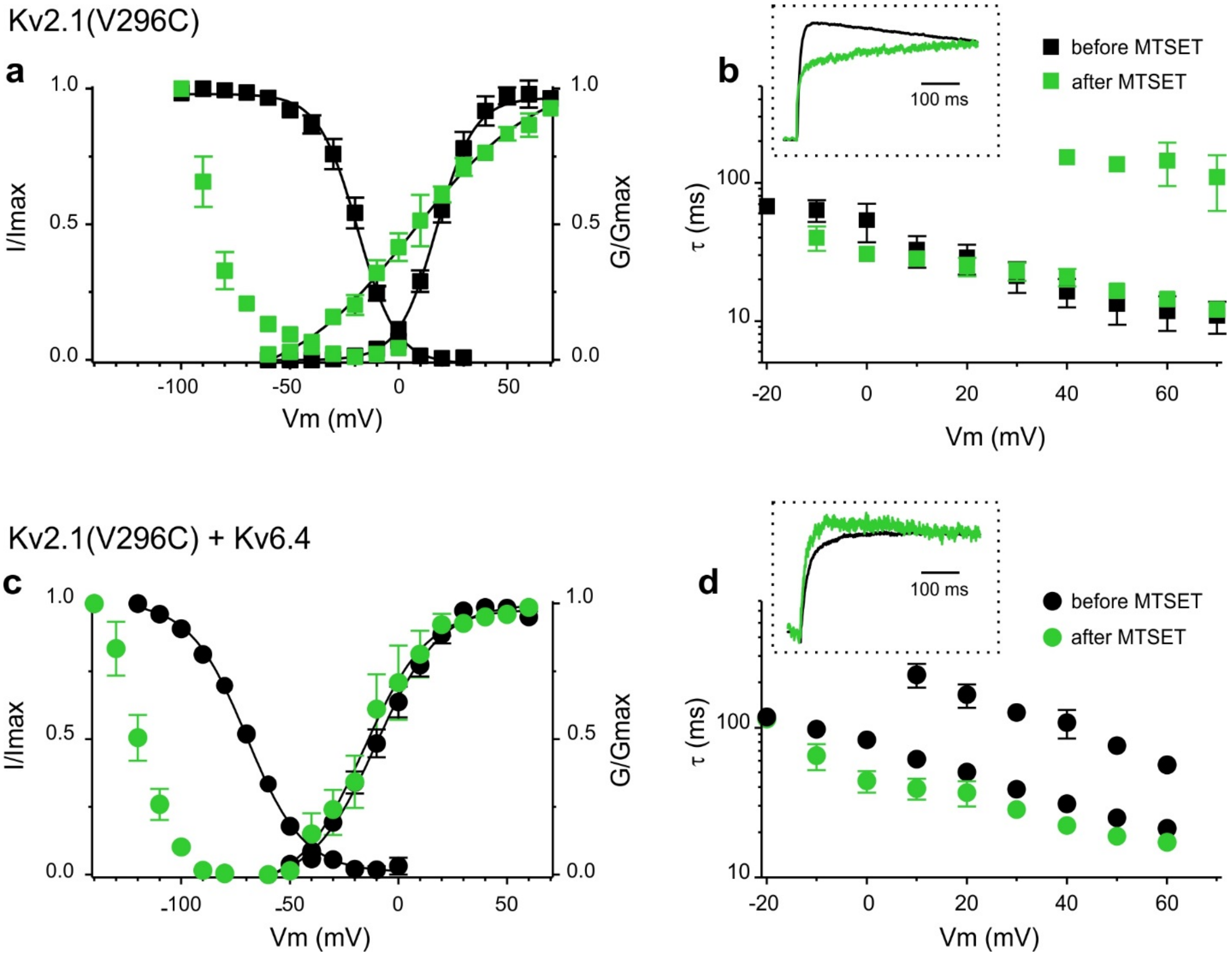

Supplementary Figure 2. Ionic current properties of Kv2.1(V296C) homotetramers and Kv2.1(V296C)/Kv6.4 heterotetramers before and after MTSET modification. (**a, c**) Voltage dependence of activation (right curves) and voltage dependence of inactivation (left curves) of Kv2.1(V296C) (**a**) and Kv2.1(V296C)/Kv6.4 (**c**) before (black) and after (green) MTSET modification. The activation curves were determined by plotting the normalized tail currents at -45 mV as a function of a 500 ms prepulse potential ranging from -70 to +70 mV. The inactivation curves were determined by plotting the normalized current amplitudes at +60 mV as a function of a 5 s prepulse potential ranging from -140 to +30 mV. Solid lines represent Boltzmann fits. The

best-fit parameter values for $V_{1/2}$ and k of the voltage dependence of activation were 18.3 mV and 9.2 before and -1.9 mV and 20.4 after MTSET modification, respectively for K_V2.1(V296C) and -8.8 mV and 10.8 before and -11.3 mV and 10.4 after MTSET modification, respectively for K_V2.1(V296C)/K_V6.4. The best-fit parameter values for $V_{1/2}$ and k of the voltage dependence of inactivation before MTSET modification were -19.3 mV and 9.0, respectively, for K_V2.1(V296C) and -69.1 mV and 13.6, respectively, for K_V2.1(V296C)/K_V6.4. After MTSET modification, our data on inactivation do not approach an asymptote at negative potentials so no Boltzmann fit was performed. (**b, d**) Time constants of activation of K_V2.1(V296C) (**b**) and K_V2.1(V296C)/K_V6.4 (**d**) derived from double and single exponential fits of current recordings before (black) and after (green) MTSET modification. Insets in both panels display normalized representative current traces to +60 mV. Clearly, in the case of K_V2.1(V296C) channels (**b**) complete modification produced an activation kinetic composed of two exponential components (particularly at potentials > +60 mV). The opposite was observed with K_V2.1(V296C)/K_V6.4 (**d**). All data shown were obtained from experiments in which voltage protocols were applied before and after complete modification by MTSET (i.e. six exposures of 1s MTSET at +60 mV). Data are represented as the mean ± SEM (shown when it is larger than the size of the symbol).

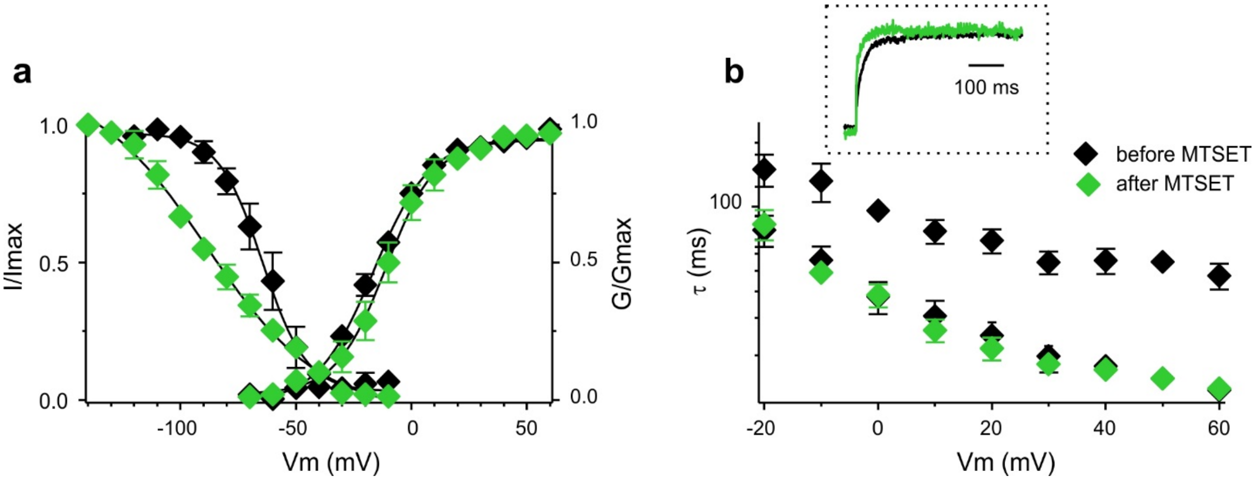

Supplementary Figure 3. Ionic current properties of K_V2.1/ K_V6.4 (V335C) heterotetramers before and after MTSET modification. (**a**) Voltage dependence of activation (right curves) and inactivation (left curves) of K_V2.1/K_V6.4(V335C) before (black) and after (green) MTSET modification. The activation and inactivation curves were determined as described in Suppl. Fig. 2a, c. Solid lines represents Boltzmann fits. The best-fit parameter values for $V_{1/2}$ and k of the voltage dependence of activation were -16.2 mV and 13.6 before MTSET modification and -14.0 mV and 12.1 after MTSET modification, respectively. The best-fit parameter values for $V_{1/2}$ and k of the voltage dependence of inactivation were -64.5 mV and 8.5 before MTSET modification and -77.3 mV and 17.2 after MTSET modification, respectively. (**b**) Time constants of activation of K_V2.1/K_V6.4(V335C) derived from double and single exponential fits of current recordings before (black) and after (grey) MTSET modification, respectively. Inset shows normalized representative current traces to +60 mV before (black) and after complete MTSET modification (green). All data shown were obtained from experiments in which voltage protocols were applied before and after complete modification by MTSET (i.e. six exposures of 1s MTSET at +60 mV).

Interestingly, in all three channel constructs (Supplementary Figures 2 & 3) chemical modification of specific voltage sensors produced a profound change in the voltage dependence of inactivation. These channels are known to exhibit closed-state inactivation, so it is conceivable that changes within the voltage sensor carries large changes in inactivation. Data are represented as the mean ± SEM (shown when it is larger than the size of the symbol).
